# Role of PPAR*α* in Hepatic Carbohydrate Metabolism

**DOI:** 10.1155/2010/572405

**Published:** 2010-09-30

**Authors:** Annelies Peeters, Myriam Baes

**Affiliations:** Laboratory of Cell Metabolism, Department of Pharmaceutical Sciences, K.U.Leuven, Campus Gasthuisberg O/N2, 3000 Leuven, Belgium

## Abstract

Tight control of storage and synthesis of glucose during nutritional transitions is essential to maintain blood glucose levels, a process in which the liver has a central role. PPAR*α* is the master regulator of lipid metabolism during fasting, but evidence is emerging for a role of PPAR*α* in balancing glucose homeostasis as well. By using PPAR*α* ligands and PPAR*α* ^−/−^ mice, several crucial genes were shown to be regulated by PPAR*α* in a direct or indirect way. We here review recent evidence that PPAR*α* contributes to the adaptation of hepatic carbohydrate metabolism during the fed-to-fasted or fasted-to-fed transition in rodents.

## 1. Introduction

### 1.1. PPAR*α*

Peroxisome proliferator-activated receptor *α* (PPAR*α*) is a nuclear receptor and master regulator of lipid metabolism. In the liver of rodents, PPAR*α* is an important orchestrator of the switch from the fed to the fasted condition via activation of fatty acid catabolism by mitochondrial, microsomal, and peroxisomal *β*-oxidation in order to maintain energy homeostasis during fasting and to protect cells from lipid overload [1–4]. Fasting periods are characterized by increased hepatic fatty acid influx, which bears similarities with high fat feeding. Moreover, PPAR*α* functions as a fatty acid sensor and mediates the remodeling of hepatic lipid metabolism via the induction of several genes, like fatty acid transporters, fatty acid activation genes, and key enzymes of the fatty acid oxidation (FAO) pathways [4]. Besides inducing FAO, PPAR*α* also stimulates the synthesis of ketone bodies from fatty acids [4], which in the fasted state serve as fuel for many extrahepatic organs, such as muscle [5]. In this way, fatty acids are preferentially utilized as fuel in periods of fasting.

The transactivation potential of PPAR*α* can be stimulated either by PPAR*α* ligands or by the presence of high levels of the transcriptional coactivator peroxisome proliferator-activated receptor gamma coactivator-1*α* (PGC-1*α*), as occurs during fasting [6]. Moreover, the induction/suppression of PPAR*α* target genes will also depend on PPAR*α* expression levels which are in part autoregulated [6]. Since nonesterified fatty acids are known ligands for PPAR*α*, elevated plasma free fatty acids (FFAs) could be expected to act as endogenous PPAR*α* ligands during fasting and to mediate the fasting-induced metabolic effects. Surprisingly, it was shown that this activation of hepatic PPAR*α* does not occur by plasma FFAs [6, 7], but by fatty acids synthesized in hepatocytes *de novo* [7, 8]. Hepatic PPAR*β*/*δ* rather than PPAR*α* was shown to be responsive to elevated fasting plasma FFAs levels [6]. Moreover, also dietary fatty acids have been reported to be able to activate PPAR*α* [9–11]. 

Importantly, there are clear differences in PPAR*α* biology between mice and humans. It was long thought that in comparison with mouse liver, human liver contains 10-fold lower levels of PPAR*α* mRNA [12], but recent investigations indicated that both species contain comparable levels [13] which may fluctuate throughout the day and according to feeding status. Although the induction of mitochondrial FAO and lipid-lowering effects are universal for PPAR*α* ligands, they do not induce peroxisome proliferation or tumor development in the liver of species other than rodents [14, 15]. Using primary mouse and human hepatocytes, it was demonstrated that besides the common regulation of lipid metabolism, PPAR*α* ligands induce a divergent set of genes in both species [13]. Notably, changes in carbohydrate gene expression were only observed in mouse hepatocytes.

### 1.2. Hepatic Glucose Metabolism: From the Fed to the Fasted State and Back

Tight control of blood glucose levels is crucial, since a fall in blood glucose can cause metabolic dysfunction, brain dysfunction, seizures, coma, and death. A persistent elevation in blood glucose leads to glucose toxicity, since hyperglycemia induces tissue damage through mitochondrial superoxide production which contributes to *β*-cell dysfunction and micro- and macrovascular complications of diabetes such as neuropathies and vasculopathies [16, 17]. 

In order to protect blood glucose levels and depending on the metabolic needs, hepatic carbohydrate metabolism undergoes a shift from glucose storage via glucose uptake and glycogen synthesis during feeding towards glucose production via glycogenolysis and gluconeogenesis during fasting. In this way, the liver plays a central role in the adaptive response to fasting. During the first hours of fasting, glycemia is preserved by the process of glycogenolysis at the expense of liver glycogen reservoirs. During prolonged fasting, when glycogen stores become critically low, hepatic glucose production shifts to *de novo* glucose synthesis (gluconeogenesis) in order to maintain blood glucose levels. These metabolic changes are reflected in the expression and/or activity of hepatic enzymes. For example, prolonged fasting induces a decrease of the maximal activity of the glycolytic enzyme glucokinase and stimulates the expression of the gluconeogenic gene phosphoenolpyruvate carboxykinase (PEPCK). Simultaneously, the lipogenic enzymes acetyl-coenzyme A carboxylase and fatty acid synthase are suppressed and the activity of mitochondrial *β*-oxidation is enhanced. As a consequence, the hepatic carbon flux is directed towards gluconeogenesis and glucose output rather than glucose uptake and glycolysis, and towards FAO and ketogenesis rather than *de novo* fatty acid synthesis. During the first few hours of refeeding after prolonged fasting, this pattern of carbon flux is maintained although the gluconeogenic product glucose-6-phosphate (G6P) is directed towards glycogen synthesis, while glucose output is suppressed.

As already mentioned, PPAR*α* is activated in liver after transition from the fed to the fasted state. Although PPAR*α* has mostly been connected with fatty acid catabolism, several lines of evidence indicate that PPAR*α* has an important role in the control of glucose homeostasis as well. For a start, fasting PPAR*α* ^−/−^ mice display marked hypoglycemia (see Section 3.1) [3, 7, 18–25]. Next, several enzymes of carbohydrate metabolism were shown to be regulated by PPAR*α* (see Sections 2 and 3). Finally, PPAR*α* was proposed to have a role in influencing insulin sensitivity (see Section 4). In this multitude of studies, often, contradictory findings were published. These sometimes depended on the experimental system used, but opposing results were also obtained for comparable paradigms. Therefore, this paper focuses on the links between PPAR*α* and glucose homeostasis in rodent liver and on the potential direct and indirect mechanisms governing this regulation. 

## 2. Role of PPAR*α* in the Use of Glucose-6-Phosphate

**Figure 1:**
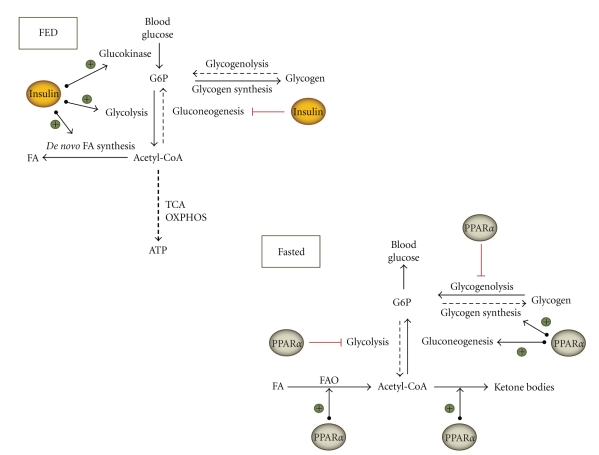
Influence of insulin and PPAR*α* on carbohydrate metabolic pathways. Different fates and different sources of hepatic G6P are depicted, together with the regulatory effects of PPAR*α*. FA: fatty acids; FAO: fatty acid oxidation; G6P: glucose-6-phosphate; OXPHOS: oxidative phosphorylation; TCA: tricyclic acid cycle.

In the fed state, when blood glucose levels are high, glucose is taken up by the liver and rapidly phosphorylated to G6P by glucokinase. G6P is at the crossroads of 4 pathways: it can be broken down via glycolysis towards pyruvate, stored as glycogen, dephosphorylated to glucose, or used in the oxidative arm of the pentose phosphate pathway (PPP) (Figure 1). 

### 2.1. Role of PPAR*α* in Regulating Glycolysis

**Figure 2:**
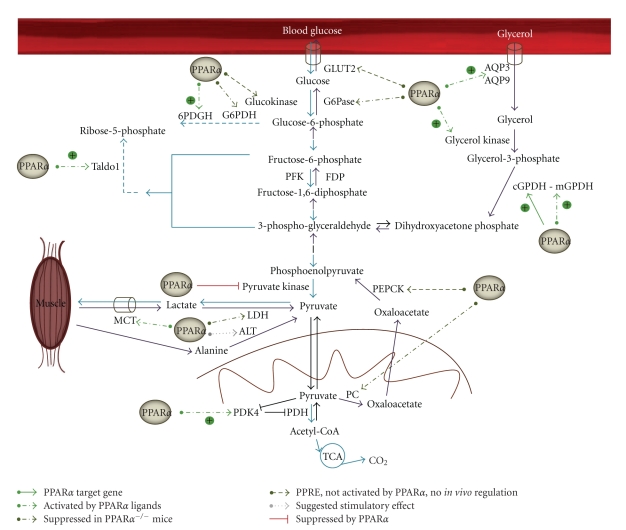
Influence of PPAR*α* on hepatic glycolysis and gluconeogenesis. The different fates of glycolytic products and gluconeogenic precursors are depicted, together with the regulatory effects of PPAR*α*. Blue arrows show breakdown of glucose via glycolysis, PPP and TCA cycle; purple arrows indicate gluconeogenic steps. Genes which were proven to be directly regulated by PPAR*α* are presented as a full green arrow. Stimulatory effects which were only proven by treatment with PPAR*α* ligands are shown as a dashed bright green arrow. An effect that was only observed in PPAR*α* ^−/−^ mice is depicted as a dark green dashed arrow. Genes in which a PPRE was identified, but no *in vivo *activation by PPAR*α* was observed are presented with a dark green dotted arrow. Genes which were only suggested to be stimulated by PPAR*α* are indicated with a grey dotted arrow. A suppressive effect of PPAR*α* is shown with a red mark. 6PDGH: 6-phosphogluconate dehydrogenase; ALT: alanine transaminase; AQP: aquaporin; FDP: fructose-di-phosphatase; G6Pase: glucose-6-phosphatase; G6PDH: glucose-6-phosphate dehydrogenase; GLUT2: glucose transporter 2; cGPDH: cytosolic glycerol 3-phosphate dehydrogenase; MCT: monocarboxylate transporter; mGPDH: mitochondrial glycerol 3-phosphate dehydrogenase; LDH: lactate dehydrogenase; PC: pyruvate carboxylase; PDH: pyruvate dehydrogenase; PDK4: pyruvate dehydrogenase kinase 4; PEPCK: phosphoenolpyruvate kinase; PFK: phosphofructokinase; Taldo1: transaldolase 1; TCA: tricyclic acid cycle.

Because PPAR*α* coordinates the fasting response in liver, it can be questioned whether it also contributes to the suppression of glycolysis. Glycolysis serves only as a small generator of ATP whereas it is the principal supplier of pyruvate, which can be further metabolized. Important players in glycolysis are transporters for glucose entry, glucokinase, and the key glycolytic enzymes phosphofructokinase (PFK) and pyruvate kinase (PK) (Figure 2). Hepatic glucokinase expression levels and glycolytic flux are mainly regulated in response to the feeding status. Glucokinase transcription is induced by insulin via sterol regulatory element-binding protein 1c (SREBP-1c) and repressed by glucagon [26]. The rate-limiting enzyme PFK is also regulated by insulin [27]. Hepatic expression of PK [28] is tuned independently of insulin, via glucose signaling, which involves binding of carbohydrate response element binding protein to the promoter. The postglycolytic fate of pyruvate is determined by pyruvate dehydrogenase kinase 4 (PDK4), which phosphorylates and inactivates pyruvate dehydrogenase (PDH), the mitochondrial enzyme needed to convert pyruvate to acetyl-coenzyme A (acetyl-CoA) (Figure 2). The latter can enter the tricarboxylic acid (TCA) cycle for energy production but can also be used to build fatty acids for energy storage (Figure 1). When PDH is inactive, pyruvate cannot enter the mitochondria and will be metabolized to lactate, in order to regain NAD^+^ units. Transcription of PDK4 is stimulated by PPAR*α* and glucocorticoids during fasting and is suppressed by insulin [29].

Administration of the PPAR*α* ligand fenofibrate to mice caused decreased expression levels of glucokinase as well as a decreased flux through this enzyme, indicative of lower hepatic glucose uptake [30]. Decreased transcripts of PK [30–33] and decreased glucokinase activity [31] after administration of an exogenous PPAR*α* ligand, suggested that PPAR*α* indeed controls the glycolytic pathway (Figure 2). In contrast, in other studies, glucokinase expression was not affected by feeding mice the PPAR*α* activator WY14643 [7]. The rat glucokinase promoter contains a functional PPAR response element (PPRE) [34], which was shown to be activated both by LXR*α*/RXR*α* and PPAR*γ*/RXR*α* in a luciferase reporter assay [26]. Interaction of this PPRE with PPAR*α*, however, was not studied so far. 

WY14643 reduced basal and glucose-stimulated PK expression in primary hepatocytes [33]. In line with these findings, clofibrate or WY14643 treatment of rats also strongly reduced PK expression [31–33] whereas this suppression did not occur in PPAR*α* ^−/−^ mice [32]. A direct interaction of PPAR*α* with the PK promoter could not be proven [32], and hence suppression of gene expression must involve some intermediary factors. It was proposed that ligand-activated PPAR*α* interferes with coactivator recruitment and decreases histone H4 acetylation of PK [33]. In conflict with these observations showing suppressed glycolytic enzymes by PPAR*α* activators, a decreased expression of glucokinase and pyruvate kinase was reported in PPAR*α* ^−/−^ mice both in fasted and in fed conditions [20, 35]. 

It is not clear why both after PPAR*α* activation and PPAR*α* depletion glycolytic enzyme levels are suppressed. In fact, both the long-term deletion of this transcription factor, which might install a new metabolic homeostasis, as the long-term activation of PPAR*α* using synthetic high affinity agonists, are nonphysiological conditions and the results have to be interpreted with care. Refeeding is expected to induce glycolytic enzymes in response to insulin signaling. This was not the case in PPAR*α* deficient mice, possibly indicating that PPAR*α* ^−/−^ hepatocytes are insulin resistant (see also below). 

Not only will activated PPAR*α* reduce the amounts of pyruvate formed, but it will also powerfully prevent the entry of pyruvate into the mitochondrial TCA cycle by strongly inducing the expression of PDK4 [36, 37]. Because PPAR*α* simultaneously stimulates *β*-oxidation, it privileges FAO to provide acetyl-CoA for the generation of energy via the TCA cycle and oxidative phosphorylation (OXPHOS). As a result, pyruvate will be available for gluconeogenesis, which implies a role for PPAR*α* in gluconeogenic regulation. Two PPREs have been identified in the promoter of PDK4 [38]. Although PDK4 expression robustly increases in response to PPAR*α* activators and fasting, PPAR*α*/RXR*α* did not bind these PPREs with high affinity in a gel-shift assay [38]. Earlier, it was already proposed that PDK4 is influenced by PPAR*α* indirectly [39]. Because the coactivator PGC-1*α* is able to bind and activate the PDK4 promoter, it is believed to be involved in the upregulation of PDK4 in response to fasting [40]. The essential role of PPAR*α* in the induction of PDK4 upon fasting was also confirmed by the absence of this response in PPAR*α* knockout mice [39]. Furthermore, in wild-type mice, PDK4 levels were normalized after 6 hours refeeding following a period of fasting, which caused suppression of gluconeogenic rates and stimulation of glycolysis and lipogenesis. This response was blunted as well in PPAR*α* ^−/−^ mice [39].

Summarizing, during the fed-to-fasted transition, PPAR*α* induces and orchestrates a switch from glucose to fatty acid utilization for energy production in hepatocytes. Simultaneous with the activation of FAO by PPAR*α*, glycolysis is inhibited. PPAR*α* activation reduces PK expression and induces PDK4 (Figure 2). So far, however, no direct interaction of PPAR*α* with the promoters of these genes was demonstrated.

### 2.2. Role of PPAR*α* in Regulating the Pentose Phosphate Pathway

Interestingly, it was suggested that the flux through the PPP was increased after fenofibrate treatment [30]. Indeed, expression levels of 6-phosphogluconate dehydrogenase (6PDGH), the rate-limiting enzyme of the oxidative branch of the PPP, and transaldolase 1 (Taldo1), a key enzyme for regulating flux from the triose phosphate pool through the nonoxidative limb of the pentose cycle, were augmented in fenofibrate treated mice [30] (Figure 2). This was in line with reduced levels of several PPP enzymes in PPAR*α* ^−/−^ mice [35]. Furthermore, the reduced content of G6P in fenofibrate-treated mice is difficult to reconcile with an increased flux through gluconeogenesis (see below) and with reduced glycolysis (see above). Therefore, the hypothesis of increased rates of glucose oxidation through PPP is attractive. PPP remodeling of hepatic glucose metabolism upon PPAR*α* activation may be important to provide NADPH needed to maintain the lipogenic flux, but may also support antioxidant action, since PPP is coupled to the synthesis of reduced gluthatione [41].

### 2.3. Role of PPAR*α* in Glycogen Homeostasis

When nutrient supply is abundant, excess glucose is stored as glycogen in liver and muscle. While skeletal muscle glycogen mainly serves to fuel muscle contractions, hepatic glycogen stores are used to keep blood glucose levels up to the mark between meals. 

**Figure 3:**
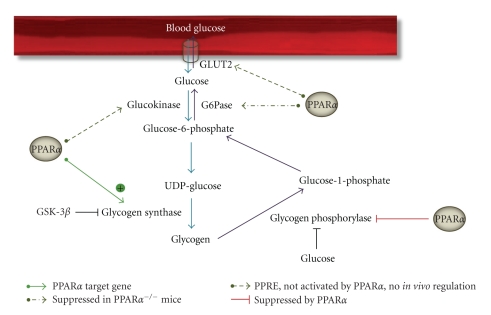
Influence of PPAR*α* on hepatic glycogen metabolism. Synthesis (blue arrows) and breakdown of glycogen (purple arrows) are depicted, together with the regulatory effects of PPAR*α*. Direct transcriptional influence is presented as a full green arrow. An effect that was only observed in PPAR*α* ^−/−^ mice is depicted as a dark green dashed arrow. Genes in which a PPRE was identified, but no *in vivo *activation by PPAR*α* was observed are presented with a dark green dotted arrow. A suppressive effect of PPAR*α* is shown with a red mark. G6Pase: glucose-6-phosphatase; GLUT2: glucose transporter 2; GSK-3*β*: glycogen synthase kinase 3*β*; UDP-glucose: uridine diphosphate glucose.

Glycogen synthase 2 (Gys-2) is the rate-limiting enzyme in the synthesis of glycogen in liver (Figure 3). The activity of Gys-2 is inhibited via phosphorylation by glycogen synthase kinase 3*β* (GSK-3*β*). In turn, GSK-3*β* kinase activity is attenuated after phosphorylation by Akt. Glycogen storage in liver is controlled by many factors, of which the rate of portal venous glucose delivery to the liver and insulin levels are best known. Insulin stimulates glycogen synthesis via Akt-mediated phosphorylation of GSK-3*β*, while glucagon inhibits Gys-2 activity through cAMP-mediated activation of GSK-3*β*. In periods of food deprivation, hepatic glycogen is broken down in order to maintain euglycemia. Glycogen phosphorylase (GP), the key enzyme responsible for glycogenolysis, generates glucose-1-phosphate, which is converted to G6P by phosphoglucomutase (Figure 3). Glucose-6-phosphatase (G6Pase), which catalyzes the conversion to glucose, is the rate-limiting enzyme in the regulation of blood glucose levels by breakdown of glycogen. The activity of GP is mainly inhibited by the presence of high levels of glucose.

Remarkably, during the first hours of fasting, when PPAR*α* levels increase, hepatic Gys-2 expression and activity are induced simultaneously with the active breakdown of glycogen stores [21, 42–44]. It was suggested that this may serve to prime the glycogen synthesizing system to rapidly replenish stores when dietary glucose becomes available again [44].

Gys-2 was identified to be a PPAR*α* target gene since PPAR*α* ligands induced Gys-2 expression in rat and mouse primary hepatocytes but not in PPAR*α* ^−/−^ hepatocytes [44]. Two putative PPREs were identified in the mouse *Gys-2* gene. Based on chromatin immunoprecipitation (ChIP) analysis, gel-shift experiments and luciferase reporter assays, the direct repeat 1 (DR1) in intron 1 (DR1int) was shown to be the response element for PPARs and the DR1 in the upstream promoter (DR1prom) the response element for hepatocyte nuclear factor 4 alpha (HNF4*α*). In liver, which expresses high amounts of HNF4*α*, DR1prom is occupied and activated by HNF4*α*, but not by PPAR*α*, while DR1int is bound by PPAR*α* and not by HNF4*α* [44]. It was suggested that during fasting, when hepatic PPAR*α* levels increase, competition between these two transcription factors may take place at the level of binding to common co-activator proteins [44]. Taken together, these* in vitro* observations indicate that besides HNF4*α*, PPAR*α* activation might promote Gys-2 expression. For GP, the key enzyme of glycogen breakdown, there are no *in vitro* studies on regulation by PPAR*α* so far.

A number of studies were conducted in which PPAR*α* agonists were administered to rodents after which glycogen content, glycogen metabolizing enzyme expression, and/or fluxes were monitored. In all cases, a reduction of hepatic glycogen storage was seen but the proposed underlying mechanisms are rather contradictory. In mice maintained on a diet containing ciprofibrate or fenofibrate, lower hepatic glycogen stores [25, 30] were accompanied by lower hepatic G6P content [30], which is both the precursor in glycogen synthesis and the end product of glycogen breakdown. According to Oosterveer et al., there was both an increased flux of glycogen synthesis through Gys-2 and an increased flux of glycogen breakdown through GP [30]. In contrast, clofibrate treatment of rats reduced both Gys-2 and GP activity, resulting in reduced hepatic glycogen content, however, without altering hepatic G6P levels [31]. In yet another study, treatment of rats with WY14643 did not alter Gys-2 expression, but did decrease GP expression levels [45]. Unfortunately, in none of these studies Gys-2 expression, glycogen levels, and glycogenic flux were studied simultaneously in the same model.

The findings in PPAR*α* knockout mice make the picture even more confusing. Fasted PPAR*α* ^−/−^ mice suffer from severe hypoglycemia starting already several hours after food withdrawal [3, 23]. During the first hours of fasting, blood glucose levels are mostly maintained by hepatic glycogenolysis. The steeper drop in blood glucose in PPAR*α* ^−/−^ mice during early fasting, therefore, may reflect a reduced glycogen reserve or impaired liberation of glucose. Several groups reported that in the fed state hepatic glycogen stores were lower in PPAR*α* ^−/−^ mice [3, 20, 21] compared to control mice, but no difference was observed in the fasted state [3, 20]. On the other hand, a study performed by Bandsma et al. [21] showed a reduced depletion of glycogen upon fasting (15 h or 24 h) concomitant with lower GP levels. According to several other groups, both hepatic glycogen storage in the fed state [25, 39] and glycogen depletion in response to fasting [20, 25, 39] were unaffected in PPAR*α* ^−/−^ mice. It is not clear why opposing results were obtained by different investigators using the same mouse model. 

More consistent results were obtained in studies where a prolonged period of fasting was followed by refeeding. Repletion of glycogen stores via hepatic glycogen synthesis was consistently impaired by PPAR*α* deficiency in all studies [20, 39, 44], despite normal insulin and glucose levels after refeeding [39]. It was proposed to be the consequence of reduced FAO and subsequently reduced gluconeogenesis and glycogen synthesis due to hampered production of substrates [39]. In the absence of PPAR*α*, expression of Gys-2 was markedly reduced during refeeding after prolonged fasting [44], likely explaining the diminished rate of glycogen formation upon refeeding in PPAR*α* ^−/−^ mice.

Notably, despite reduced Gys-2 levels, gluconeogenic flux in fasted PPAR*α* ^−/−^ mice was directed more towards glycogen, than towards blood glucose output [21]. However, no explanation was given for this discrepancy. Expression of GP was suppressed in fasted PPAR*α* ^−/−^ mice, but regrettably, the flux through GP was not studied [21].

Apart from a direct effect on glycogen metabolizing enzymes, PPAR*α* may indirectly affect the fate of newly formed G6P which is either used in glycolysis, for transfer to plasma as glucose, for glycogen synthesis or to enter the PPP pathway (Figure 1). Normally, insulin directs newly formed G6P towards glycogen disposition. According to Sugden et al., PPAR*α* deficiency resulted in impaired insulin action (see also below) with respect to net hepatic glycogen disposition starting from G6P on refeeding after starvation [39], resulting in slower repletion of glycogen stores. In sharp contrast, as mentioned above, it was shown by performing flux studies with stable isotopes that newly synthesized G6P was partitioned away from plasma glucose towards glycogen synthesis in PPAR*α* knockout mice [21]. These two lines of observations are mutually exclusive. However, the observation of reduced glycogen repletion upon refeeding and reduced Gys-2 expression levels in PPAR*α* ^−/−^ mice is consistent between the different studies, pointing to an important role of PPAR*α* in the control of glycogen synthesis (Figure 3).

## 3. Gluconeogenesis

After prolonged fasting, blood glucose for consumption by the brain, the kidney medulla, and red blood cells is exclusively maintained by gluconeogenesis that primarily takes place in hepatocytes. The main precursors for hepatic gluconeogenesis are pyruvate, lactate, amino acids, and glycerol (derived from the backbone of triglycerides), which are converted to glucose via a series of reactions in the cytosol and mitochondria (Figure 2). After depletion of carbohydrate reserves, triacylglycerol stores are mobilized from adipose tissue, increasing FFAs and glycerol concentration in plasma. These fatty acids are taken up by the liver, where they can be stored or metabolized via *β*-oxidation to produce acetyl-CoA. In turn, acetyl-CoA can be further metabolized in mitochondria via the TCA cycle followed by OXPHOS or it can be converted to pyruvate. Glycerol, pyruvate, and ATP are then used in hepatic gluconeogenesis. The main portion of lactate is produced in a process known as the Cori cycle, in which lactate produced by glycolysis in exercising muscle is shuttled to the liver where it is converted back into glucose. Also in adipose tissue, glucose is metabolized to lactate, which can subsequently be transported to the liver. Alternatively, pyruvate produced in muscle by glycolytic oxidation of glucose can be converted to alanine via transamination (see Section 3.4). Alanine is exported from muscle tissue to liver, where it is reconverted to pyruvate and subsequently used in gluconeogenesis to produce glucose. The latter can be transported back to muscle in the glucose-alanine cycle. 

Crucial steps in gluconeogenesis are the conversion of pyruvate to oxaloacetate catalyzed by pyruvate carboxylase (PC); the conversion of oxaloacetate to phosphoenolpyruvate catalyzed by PEPCK; the rate-limiting step catalyzed by fructose-1,6-bisphosphatase (FBP) (Figure 2). The final step, hydrolysis of G6P to glucose by G6Pase, is shared with the glycogenolytic pathway. PGC-1*α* is an important transcriptional coactivator in the control of gluconeogenic genes and it is strongly induced in the liver of fasting mice. Glucagon, glucocorticoids, and adrenaline induce hepatic glucose output during starvation via increasing PGC-1*α* levels and activating gluconeogenesis. The concentration of gluconeogenic substrates will also determine glucose production. During the fasted-to-fed transition, insulin suppresses PGC-1*α* mRNA levels and subsequently reduces gluconeogenic rates and glucose output.

Given the important role of PPAR*α* in the adaptive response to fasting, it is conceivable that activated PPAR*α* directly regulates or indirectly influences the expression or activity of some key gluconeogenic genes. Several *in vitro* studies have been conducted to test whether gluconeogenic genes such as PEPCK, G6Pase, and cytosolic glycerol 3-phosphate dehydrogenase (cGPDH) are potential PPAR*α* target genes. After discussion of the hypoglycemic phenotype of PPAR*α* ^−/−^ mice, the general gluconeogenic pathway and gluconeogenesis from the major substrates lactate/pyruvate will be reviewed, whereafter glucose synthesis from the minor substrates glycerol and alanine are discussed. 

### 3.1. PPAR*α* ^−/−^ Mice Are Hypoglycemic

It has been reported repeatedly that although the general appearance of PPAR*α* ^−/−^ mice is normal in the fed state [18, 19, 21, 23], they develop severe hypoglycemia during fasting [3, 7, 18–25, 35]. However, discrepancies with regard to time of onset of hypoglycemia and plasma insulin levels have been published. Hypoglycemia occurred already after a few hours of fasting according to some investigators [3, 23] whereas in other studies, normal blood glucose levels were observed after a 15-hour [21] or even 24-hour fasting period [39]. Sometimes, hypoglycemia was accompanied by increased plasma insulin concentrations as compared to wild type mice [7, 23]. In other studies, PPAR*α* ^−/−^ mice had lower blood glucose values but unaltered insulin levels in comparison with wild type mice [20, 21]. It was even reported that steady-state fasting glucose levels were normal, while fasting plasma insulin concentrations were increased in PPAR*α* ^−/−^ mice [39]. It is not resolved why opposing results were obtained when studying the same knockout mice.

Several different mechanisms were put forward to explain the fasting-induced hypoglycemia in PPAR*α* ^−/−^ mice, some of which may be operative simultaneously. Some researchers believe this is mainly due to hepatic defects including liver glycogen depletion [3, 18], a blunted gluconeogenic response [18–20, 22, 24], reduced FAO rates [3, 18, 20, 25] and/or stronger inhibition of hepatic glucose output due to partitioning of G6P away from blood glucose [21]. Others blame increased glucose utilization in extrahepatic tissues [3, 20, 23, 35]. In favour of the last option is the fact that hepatic reexpression of PPAR*α* did not rescue the metabolic phenotype of PPAR*α* ^−/−^ mice [23].

### 3.2. Gluconeogenesis from Lactate/Pyruvate

When lactate is used as a precursor of *de novo* glucose synthesis, it first has to be converted to pyruvate by lactate dehydrogenase (LDH). Subsequently, PC, which is localized in mitochondria, catalyzes the conversion of pyruvate to oxaloacetate, which is a substrate for PEPCK, one of the key enzymes in gluconeogenesis (Figure 2). 

PPAR*α* was shown to activate PEPCK promoter activity in Hepa1c1c7 cells [46], and in isolated rat hepatocytes, PEPCK and G6Pase expression increased in response to palmitate [47]. The PEPCK promoter contains 2 intermediate-affinity PPREs [48]. These PPREs were responsible for the upregulation of PEPCK by PPAR*γ* in adipocytes [49], but direct interaction with PPAR*α* was not studied. ChIP experiments showed that upon palmitate treatment, PPAR*α* was recruited to the G6Pase promoter together with HNF4*α* and several other transcription factors [47]. Sites of interaction with the G6Pase promoter region or functional studies, however, were not reported. No PPRE has been identified in the promoter of LDH or PC [4].

When rodents were treated with PPAR*α* ligands, the effects on gluconeogenesis were rather variable. In rats, G6Pase activity was not affected [31] whereas PEPCK expression levels were reduced [50] and LDHb levels were increased [45]. WY14643 feeding of mice had no effect on PC, PEPCK or G6Pase expression levels [7]. In a more informative study, mice were treated with fenofibrate and not only expression levels of individual enzymes but also gluconeogenic flux was analysed [30]. This flux was increased which was mirrored by increased expression of glycerol kinase (see also Section 3.3), but expression of PGC-1*α*, PEPCK and G6Pase was unaltered [30].Interestingly, the induction of the gluconeogenic genes PEPCK and G6Pase by the glucocorticoid dexamethasone was shown to be PPAR*α*-dependent both in mice as well as in isolated human hepatocytes [51]. Lactate, being the dominant gluconeogenic precursor, and which is mainly derived from the Cori cycle, is transported into hepatocytes via the monocarboxylate transporter (MCT). Hepatic MCT-1 expression was shown to be upregulated by fasting, WY14643, fenofibrate and dietary PPAR*α* agonists in mice and rats [52, 53], resulting in increased supply of gluconeogenic substrates.

Transcript levels of key gluconeogenic enzymes were not uniformly altered in PPAR*α* knockout mice. PEPCK expression levels were shown to be similarly induced upon fasting in wild-type and PPAR*α* ^−/−^ mice [3, 22], which suggests that PEPCK is upregulated by fasting in a PPAR*α* independent manner. Yet, lower expression of PEPCK in 24 hours fasted [21] and refed [20] PPAR*α* ^−/−^ mice in comparison with control animals was reported as well. G6Pase and G6P translocase were not differently expressed between fed PPAR*α* ^−/−^ and wild type mice, but induction upon fasting of these two enzymes was impaired in PPAR*α* ^−/−^ mice [21]. Furthermore, a severe decrease in expression of LDH and PC was found in PPAR*α* ^−/−^ mice [4, 35], which suggests impaired gluconeogenesis from lactate. However, most groups reported normal plasma lactate levels in PPAR*α* ^−/−^ mice [20, 21, 25]. This suggests a supportive role for PPAR*α* in the gluconeogenic response to fasting (Figure 2).

More important than expression levels of gluconeogenic enzymes is the capacity to produce glucose. In a study with isolated hepatocytes from PPAR*α* ^−/−^ mice, glucose production from pyruvate was not significantly altered [23], but this was contradicted by Le May et al. who found a 20% reduction in gluconeogenesis from lactate/pyruvate in PPAR*α* ^−/−^ hepatocytes [24]. The rate of *de novo* synthesized G6P and hepatic G6P levels were not different in 15 hours fasted PPAR*α* ^−/−^ mice as compared with wild type mice [21]. However, the rate of G6P towards plasma glucose was diminished, while synthesis of uridine diphosphate glucose (UDP-glucose), and thus glycogen formation was higher [21], which could contribute to fasting hypoglycemia. This was in line with decreased hepatic glucose production (HGP) in fasted PPAR*α* ^−/−^ mice [22]. The regulation of HGP from pyruvate/lactate in PPAR*α* ^−/−^ mice during the physiologic situation of a moderate overnight fast (17 hours) and refeeding (5 hours) was studied more in detail using ^13^C-mass isotopomer distribution analysis (MIDA) by Xu et al. [20]. In PPAR*α* ^−/−^ mice, decreased HGP from lactate was observed in the fed as well as in the fasted state [20]. 

Most studies mainly observed metabolic alterations in PPAR*α* ^−/−^ mice in starved conditions [3, 18–24], whereas fed PPAR*α* ^−/−^ mice had normal blood glucose [18, 19, 21, 23]. In a study performed by Atherton et al., however, metabolic alterations were also observed in fed PPAR*α* ^−/−^ mice [54]. Here, metabolic profiling of several PPAR*α* ^−/−^ tissues was performed via ^1^H-NMR spectroscopy and MS. Important metabolic differences were detected in all tissues, but in particular in liver. Liver of PPAR*α* ^−/−^ mice contained profoundly decreased levels of glucose, several amino acids including glutamine and alanine, and increased levels of lactate. The combined presence of lower glucose and higher lactate content confirms impaired gluconeogenesis from lactate in PPAR*α* ^−/−^ mice. This demonstrates that a failure to express PPAR*α* results in perturbed balance between glycolysis, TCA cycle and gluconeogenesis [54]. 

Since the fasted PPAR*α* ^−/−^ liver fails to induce fatty acid *β*-oxidation, ketone body synthesis is hampered and peripheral organs cannot be fueled with ketone bodies [3, 18, 19, 21, 25, 39]. Therefore, these organs solely rely on glycolytic consumption of glucose and increased amounts of lactate arise. However, since expression of key gluconeogenic genes and HGP are not increased in PPAR*α* ^−/−^ mice compared with control mice [3, 20, 22, 35], this suggests that the PPAR*α* ^−/−^ liver does not upregulate gluconeogenesis via compensatory mechanisms despite decreased systemic glucose levels and increased peripheral production of the gluconeogenic substrate lactate.

### 3.3. Gluconeogenesis from Glycerol

Cellular uptake of glycerol is mediated by the transporters aquaporin 3 and 9 (AQP3 and AQP9). When glycerol is used as gluconeogenic precursor, this is first phosphorylated to glycerol 3-phosphate via the action of glycerol kinase. This in turn is converted to the gluconeogenic intermediate dihydroxyacetone phosphate via cytosolic (cGDPH) or mitochondrial glycerol 3-phosphate dehydrogenase (mGDPH) (Figure 2).

The promoter of cGPDH was shown to contain a functional PPRE by promoter deletion studies and was functionally identified to be a direct target of PPAR*α* by transactivation, gel shift and ChIP experiments [22].

Treatment of mice with fenofibrate resulted in increased expression of glycerol kinase [30]. Fasting as well as treatment with WY14643 induced an upregulation of the expression of genes involved in hepatic gluconeogenesis from glycerol, including AQP3, AQP9, cGDPH, mGDPH, and glycerol kinase [22], which was dependent on the presence of PPAR*α* (Figure 2). In line with the upregulation of glycerol utilization genes via PPAR*α*, WY14643 significantly decreased plasma glycerol levels in wild type but not in PPAR*α* ^−/−^ mice [22]. This decrease in plasma glycerol levels was also observed in human atherosclerotic patients treated with fenofibrate for 4 weeks [22]. 

In accordance, decreased HGP from glycerol was observed in fasted PPAR*α* ^−/−^ mice [22]. However, opposite findings were reported by Xu et al. [20], including enhanced glycerol production, enhanced HGP from glycerol, and enhanced total HGP in 17-hour fasted PPAR*α* ^−/−^ mice, which was suggested to occur as a compensating mechanism for the decreased HGP from lactate. 

Depending on the nutritional status, the importance of glycerol as gluconeogenic precursor varies from 5% postprandial in humans to being the main gluconeogenic precursor after prolonged starvation in rodents [22]. Inasmuch as the conversion of glycerol to glucose in liver is impaired in PPAR*α* ^−/−^ mice, defective synthesis of glucose from glycerol may partly explain the fasting-induced hypoglycemia in PPAR*α* ^−/−^ mice. In conclusion, the metabolic fate of glycerol is under the control of PPAR*α*, which stimulates its conversion to glucose in liver (Figure 2).

### 3.4. Gluconeogenesis from Alanine

During fasting, one of the mechanisms for the synthesis of glucose involves protein degradation, followed by the production and export of alanine from muscle tissue to liver. Alanine is metabolized by deamination and gluconeogenesis in the liver in the glucose-alanine cycle [55]. Since hepatic levels of alanine were lower in PPAR*α* ^−/−^ mice, it was suggested that the enzymes controlling this cycle may be constitutively more active in PPAR*α* ^−/−^ mice [54]. It should be noted that PPAR*α* has a more general suppressive effect on the trans- and deamination of amino acids in rodents [56].

### 3.5. Gluconeogenesis—Conclusion

Upon activation of PPAR*α*, FAO is stimulated and increased amounts of acetyl-CoA are produced. Further metabolism via TCA cycle and OXPHOS provides the liver with intermediates and energy for gluconeogenesis in order maintain fasting euglycemia.

PPAR*α* receptor function regulates the rate and route of HGP in the fasted state by controlling the flux of lactate and glycerol to glucose (Figure 2). Although not all studies are in agreement, a dual block in the early steps of the conversion of lactate to glucose and glycerol to glucose in PPAR*α* ^−/−^ mice causes a maladaptation to fasting and may at least partly explain the development of hypoglycemia in these mice.

cGPDH is the only gluconeogenic gene which was identified to be a direct PPAR*α* target gene. Although PPAR*α* plays a role in fasting-induced stimulation of gluconeogenic gene expression, this may occur via supporting glucocorticoid action rather than direct stimulation of gene transcription.

## 4. PPAR*α* and Hepatic Insulin Sensitivity

Whole body insulin sensitivity is the result of both peripheral and hepatic insulin action [35]. Peripheral action of insulin affects tissue glucose uptake, and therefore glucose clearance. In liver, insulin affects the net balance of gluconeogenic versus glycolytic flux by influencing gene expression levels.

It was shown that PPAR*α* has an important modulatory function on hepatic insulin action through its target TRB3, the mammalian *tribbles* homolog. During fasting and upon WY14643 treatment, hepatic expression of TRB3 was induced in wild type, but not in PPAR*α* ^−/−^ mice [57]. TRB3 disrupts insulin signaling by interfering with activation of Akt [58]. The PPAR*α*-mediated induction of TRB3 was proposed to suppress insulin action, to induce insulin resistance, and subsequently to promote gluconeogenesis [57].

Based on these findings, it was expected that in the absence of PPAR*α*, insulin sensitivity would be increased. However, in sharp contrast, hepatic insulin resistance was observed in PPAR*α* ^−/−^ mice. 

Insulin action is of particular importance in liver during the fasted-to-fed transition. Insulin then stimulates glycogen repletion, glycolysis, and hepatic lipogenesis and suppresses hepatic PDK4 protein expression and HGP. In PPAR*α* ^−/−^ mice, it was observed that all these insulin actions were impaired, and therefore these mice were denoted as insulin resistant [20, 35, 39, 44]. For example, differential expression between the fasted and fed state of several insulin-responsive genes was lost in PPAR*α* ^−/−^ mice [35]. Both in the fed and fasted state, G6P dehydrogenase (G6PDH) and Taldo expression levels were lower in PPAR*α* ^−/−^ mice and during refeeding, induction of glucokinase expression was blunted [35]. Because SREBP-1c is a major mediator of insulin action on hepatic glycolytic and lipogenic gene expression [59], lower SREBP-1c expression levels in liver of PPAR*α* ^−/−^ mice also point to reduced hepatic insulin sensitivity. 

Summarizing, during the fasted state, PPAR*α* may have a stimulatory role on gluconeogenesis not only via direct gene induction or supporting glucocorticoid action (see Section 3.2), but also through impairment of insulin signaling, by inducing TRB3.

On the other hand, PPAR*α* deficiency severely reduced the responsiveness to insulin during the fasted to fed transition in terms of gluconeogenic, glycolytic, and lipogenic enzyme expression. The mechanism of this hepatic insulin resistance in PPAR*α* knockout mice was not resolved, but it is likely not related to TRB3 and rather caused by fatty acid accumulation as a result of impaired FAO.

## 5. Glucose as PPAR*α* Activator

Besides the previously discussed modulatory role of PPAR*α* on carbohydrate pathways in liver, recently, a new and direct link between PPAR*α* and glucose has been proposed. 

Hostetler et al. [60] showed that both glucose and its metabolites glucose-1-phosphate and G6P are endogenous ligands of PPAR*α* with glucose having the highest affinity, well within the range of normal physiological levels. These metabolites are supposed to interact directly with specific amino acid residues in the PPAR*α* ligand binding domain [60], resulting in altered PPAR*α* secondary structure [60, 61]. After glucose binding, coactivator recruitment, DNA binding and transactivation potential of PPAR*α*/RXR*α* heterodimers were increased [60], but only in the presence of activating PPAR*α* ligands such as arachidonic acid or clofibrate. In the absence of other ligands, glucose inhibited recruitment of coactivators and therefore suppressed PPAR*α* regulated transcription [60]. 

The impact of this direct interaction of glucose with PPAR*α* on the regulation of carbohydrate metabolism in physiological conditions remains to be proven. Notably, in the fasted state, when PPAR*α* is activated, hepatic glucose levels are expected to be rather low. It also remains unexpected that a hydrophilic ligand can affect a hydrophobic binding pocket. Therefore, these data await confirmation and need to be considered with care.

## 6. Concluding Remarks

From the above, it appears that hepatic PPAR*α* plays an important role in carbohydrate handling in rodent liver by regulating expression of genes in a direct or indirect way. During the fed to fasting transition, PPAR*α* activation contributes to the inhibition of glycolysis and to the induction of gluconeogenesis. However, since only a few genes were shown to be directly targeted by PPAR*α*, these effects are mostly governed by indirect mechanisms that remain to be clarified. Together with its well-known stimulatory effect on *β*-oxidation, a switch occurs from glucose to FA as the primary fuel source. PPAR*α* also impacts on the balance between glycogen synthesis and glycogenolysis. Although PPAR*α* activation is associated with a reduction in glycogen stores, it remains controversial how this is precisely established. 

When considering all published data as a whole, it is striking that experiments using PPAR*α* ligands do not lead to the same conclusions as those with PPAR*α* knockout mice. In these mice, however, a whole new metabolic homeostasis may be installed which hampers distinction between direct and indirect effects of the absence of this transcription factor. Similar remarks have to be made when organisms are treated for long periods with synthetic agonists. Other cautionary notes are that expression levels of metabolic enzymes are not always predictive of pathway fluxes, and that there is only partial overlap of genes upregulated by PPAR*α* during fasting and genes upregulated by synthetic PPAR*α* agonists [6, 9]. Therefore, the most reliable system to judge the role of PPAR*α* in carbohydrate metabolism is probably an acute activation of the transcription factor using physiological PPAR*α* ligands.

## Abbreviations

6PDGH: 6-phosphogluconate dehydrogenase
acetyl-CoA: Acetyl-coenzyme A
AQP: Aquaporin
cGPDH: Cytosolic glycerol 3-phosphate dehydrogenase
ChIP: Chromatin immunoprecipitation
DR1: Direct repeat 1
FAO: Fatty acid oxidation
FBP: Fructose-1,6-bisphosphatase
FFAs: Free fatty acids
G6P: Glucose-6-phosphate
G6Pase: Glucose-6-phosphatase
G6PDH: Glucose-6-phosphate dehydrogenase
Glut2: Glucose transporter 2
GSK-3*β*: Glycogen synthase kinase 3*β*
GP: Glycogen phosphorylase
Gys-2: Glycogen synthase 2
HGP: Hepatic glucose production
HNF4*α*: Hepatocyte nuclear factor 4 alpha
LDH: Lactate dehydrogenase
MCT: Monocarboxylate transporter
OXPHOS: Oxidative phosphorylation
PC: Pyruvate carboxylase
PDH: Pyruvate dehydrogenase
PDK4: Pyruvate dehydrogenase kinase 4
PEPCK: Phosphoenolpyruvate carboxykinase
PFK: Phosphofructokinase
PGC-1*α*: Peroxisome proliferator-activated receptor gamma coactivator-1*α*
PK: Pyruvate kinase
PPAR*α*: Peroxisome proliferator activated receptor *α*
PPP: Pentose phosphate pathway
PPRE: PPAR response element
SREBP-1c: Sterol regulatory element binding protein 1c
Taldo: Transaldolase
TCA: Tricarboxylic acid
TRB3: The mammalian *tribbles* homolog 3
UDP-glucose: Uridine diphosphate glucose.
